# Trans-replicase helper activity of porcine circoviruses promotes the synergistic replication of torque teno virus

**DOI:** 10.3389/fmicb.2024.1326696

**Published:** 2024-01-23

**Authors:** Marvin Ssemadaali, Md-Tariqul Islam, Wenjuan Fang, Zeinab Aboezz, Brett Webb, Sheela Ramamoorthy

**Affiliations:** ^1^Department of Microbiological Sciences, North Dakota State University, Fargo, ND, United States; ^2^Department of Microbiology and Immunology, Faculty of Veterinary, Animal, and Biomedical Sciences, Sylhet Agricultural University, Sylhet, Bangladesh; ^3^Department of Virology, Faculty of Veterinary Medicine, Benha University, Banha, Egypt; ^4^Veterinary Diagnostic Laboratory, North Dakota State University, Fargo, ND, United States

**Keywords:** torque teno virus (TTV), co-infection, porcine circovirus (PCV), replicase, mice, EMSA, complementation, ELISA

## Abstract

While the primary pathogenic potential of torque teno viruses (TTVs) is yet to be defined, TTVs are often co-detected with other pathogens and are suspected of exacerbating clinical disease in coinfections. Swine TTVs (TTSuVs) enhance clinical signs of porcine circovirus type 2 (PCV2) in a gnotobiotic pig model. However, the mechanisms involved are unknown. In this study, we observed that co-culture of TTSuV1 and PCV1, and specifically supplementing TTSuV1 cultures with the PCV replicase protein in trans consistently resulted in higher levels of replication of TTSuV1 when compared to TTSuV1 cultured alone. Therefore, the hypothesis that the PCV replicase (rep) protein has trans-replicase helper activity for TTSuV1 was examined. Based on EMSA and reporter gene assays, it was determined that the PCV1 rep directly interacted with the TTSuV1 UTR. The TTSuV1 rep trans-complemented a PCV rep null mutant virus, indicating that the TTSuV1 and PCV1 replicase proteins supported the replication of both viruses. In mice, the administration of plasmids encoding the PCV1 rep and a TTSuV1 infectious clone resulted in the production of higher TTSuV1 genome copies in dually exposed mice when compared to singly exposed mice. Higher sero-conversion and lymphoid hyperplasia were also observed in the dually exposed experimental mice. Thus, this study provides evidence for trans-replicase activity of PCVs and TTVs as a novel mechanism of explaining enhanced viral replication in coinfections involving both viruses.

## 1 Introduction

Torque teno viruses (TTVs) are diverse and ubiquitous commensals which colonize a wide range of mammalian species. While they were previously classified as a genus within the Circoviridae family, they were reassigned to a separate family called Anelloviridae in 2009 (Biagini, 2009; Lefkowitz et al., 2018). Whether TTVs act as primary pathogens is a topic of scientific debate. However, numerous epidemiological studies document a correlation between TTVs and a wide range of infectious and non-infectious disease conditions such as chronic hepatitis C (Kawanaka et al., 2002), autoimmunity (Rezahosseini et al., 2019), cancer (Pan et al., 2018) and respiratory disease (Bal et al., 2022). In these studies, higher TTV loads were linked to more severe diseases and immunosuppression. TTVs also contaminate blood transfusion products and are a marker for tissue rejection in organ transplantation (Karimi et al., 2013).

Among the members of the Anelloviridae family, swine TTVs (TTSuVs) are the only members with demonstrated pathogenic potential in an experimental animal models (Krakowka and Ellis, 2008). In gnotobiotic pigs, coinfection of TTSuV1 with porcine circovirus type 2 (PCV2), the cause of post-weaning multi-systemic wasting disease syndrome in piglets, or porcine reproductive and respiratory disease syndrome virus (PRRSV) resulted in enhanced clinical signs due to PCV2 and PRRSV, respectively (Ellis et al., 2008; Krakowka et al., 2008). In previous studies we found that TTSuV1 is 85% more likely to be detected in morbid pigs than healthy pigs (Rammohan et al., 2012). Given TTSuVs possible zoonotic potential (Ssemadaali et al., 2016), the detection of TTVs in pork products and their role in exacerbating coinfections (Kekarainen et al., 2009, Karimi et al., 2013, Webb et al., 2020), understanding the molecular mechanisms by which TTVs can potentially enhance other infections or disease conditions becomes important.

Like circoviruses cloned, re-circularized viral genomic DNA or dimerized infectious clones (Fenaux et al., 2002) produce TTV specific signals in cell culture and pig models. However, sustained, or robust viral replication is not achieved (Kamahora et al., 2000; Kakkola et al., 2007; Huang et al., 2012). TTVs can be readily detected by PCR in naturally infected hosts, efficient laboratory culture has been a long-standing challenge. This observation supports the premise that efficient TTV replication is supported by factors acting in trans in natural infections. These factors could originate either from the host or coinfecting agents. As an analogous model linear, single stranded adeno-associated viruses (AAV’s) are capable of independently replicating their own DNA. However, they require helper viruses such as adenovirus, herpes simplex virus and cytomegalovirus for efficient and productive replication. Promiscuous trans-replicase activity (Zhang et al., 2016) is one among several known molecular mechanisms involved in the interactions between dependoviruses and their helper viruses (Meier et al., 2020). Similarly, the replicase (rep) proteins of the non-pathogenic PCV1 and pathogenic PCV2 are highly conserved and are known to function interchangeably (Mankertz et al., 2003). Rep proteins of divergent ssDNA viral families share conserved structural features such as a nonanucleotide loop, endonuclease domains, and RCR motifs which are required for rolling circle replication of the viral genome (Mankertz et al., 2004). They even share commonalities with prokaryotic plasmid DNA replication machinery (Tarasova and Khayat, 2021). The largest TTV ORF, ORF1, encodes a multi-functional protein that serves as the capsid and also contains replicase related domains such as the rolling circle replication (RCR) motifs (Webb et al., 2020). To better understand the mechanisms by which TTSuV1 exacerbates clinical signs in pigs coinfected with TTSuV1 and PCV2 (Ellis et al., 2008; Krakowka et al., 2008), we herein explore hypothesis that the PCV rep protein has trans-replicase activity for TTSuV1 and promotes TTSuV1 replication. The findings described provide a possible mechanistic explanation for the observed increase in viral titers and pathogenicity in coinfections involving pathogenic PCVs and TTVs.

## 2 Materials and methods

### 2.1 Cells, viruses, and recombinant proteins

Porcine kidney cell line (PK15N, 005-TDV, National Veterinary Services Laboratory, Ames, IA, United States) (designated as PK15N throughout the document), PK-15 cells persistently infected with porcine circovirus type 1 (PCV1) (PK-15, CCL3-ATCC, Manassas, VA) (designated as PCV1 + PK15 cells throughout the document), and swine testicular cells (ST, ATCC, Manassas, VA) were used in the below described experiments. All reagents and PK15N and ST cells used were previously tested and found to be negative for TTSuVs and PCVs. Recombinant TTSuV1 (Gen Bank KT037083) and PCV2b 41513 (Gen Bank ALD62452) were rescued by transfection as previously described (Singh and Ramamoorthy, 2016a,b; Rakibuzzaman et al., 2021), with the exception that the genomes were dimerized to enable direct transfection and rescue without the need for excision and recircularization of the viral genome. In addition, a V5 tag encoding the amino acid sequence GKPIPNPLLGLDST was inserted into the 3′ end of the TTSuV1 ORF1 by overlap extension PCR and directional cloning to serve as a genetic and antigenic marker for the cloned TTSuV1 genomic DNA. The TTSuV1 ORF1 gene was cloned and expressed as described before (Singh and Ramamoorthy, 2016a,b). The PCV1 rep was amplified using forward primer 5′-CTGAggatgcGCCACCATGCCCAGCAAGAAGAATG-3′ and reverse primer 5′-AGCTctcgagGTAATTTATTTCATATGGAAA-3′ from the PCV1 + PK15 cell cultures. The amplified fragment was directionally cloned into a pcDNAV5-HisA mammalian expression vector (Thermo Fisher, Grand Island, NY), using the BamHI and XhoI restriction sites. The integrity of all the recombinant plasmid constructs was verified by restriction digestion and sequencing. All expressed proteins had a C-term V5 tag. Protein expression for the individual constructs was verified by transfection in PK15 and ST cells, using the TransIT-2020 (Mirus Bio, Madison, WI), following the manufacturer’s instructions and immunofluorescence assays (IFAs), using specific antibodies.

### 2.2 Virus culture

To culture TTSuV1, PK15N or ST cells were cultured to 50% confluence in 6 well tissue culture plates in the presence of DMEM containing 10% FBS and 1% penicillin streptomycin and transfected or infected as previously described (Singh and Ramamoorthy, 2016a,b; Rakibuzzaman et al., 2021). To rescue recombinant TTSuV1, the dimerized genomic TTSuV1 DNA construct was used for transfection. The PCV1 + PK-15 cells were used to prepare PCV1 cultures. Virus cultures were quantified by the TCID_50_ method and stored in aliquots at −80°C for further use. For flow cytometry cells were collected from duplicates of treatment and fixed with 4%PFA and stored at 4°C.

### 2.3 Viral detection by an immunofluorescence assay

As TTSuV1 and PCV1 are non-cytolytic, the presence of replicating virus or the expression of viral proteins was assessed by IFAs. Briefly, 50% confluent PK-15 or ST cells were infected or transfected as previously described (Singh and Ramamoorthy, 2016a,b, Rakibuzzaman et al., 2021). After 48 h of incubation, the cells were washed in Hank’s balanced salt solution (HBSS) (Corning, Manassas, VA) and fixed in ice cold acetone: methanol (1:1). Detection of the recombinant TTSuV1 virus or TTSuV1 ORF1 protein was achieved with a rabbit polyclonal hyperimmune serum collected from rabbits administered a synthetic peptide from the TTSuV1 ORF1 (RWRRRLGRRRRRYRK, position 6–20) (ProMab Biotechnologies, Richmond, CA). The specificity of the antibody was independently verified using previously a validated rabbit polyclonal anti-TTSuV1 ORF1 antibody (Huang et al., 2012) or a commercial mouse derived anti-V5 tag antibody (Thermo Fisher, Grand Island, NY). The rabbit polyclonal antibodies were used at a 1:200 dilution, and the monoclonal V5 antibody at a 1:500 dilution. The PCV1 ORF1 protein was detected using the V5 monoclonal antibody at 1:500 or polyclonal PCV1-specific swine serum at 1:200. Anti-rabbit, mouse or swine IgG conjugated to FITC (KPL, Gaithersburg, MD) was used as the secondary antibody at a 1:100 dilution for 45 min and slides were visualized in a fluorescent microscope.

### 2.4 Transfection of TTSuV1 infectious clone in PCV1 + PK15 cells

**Figure 1:**
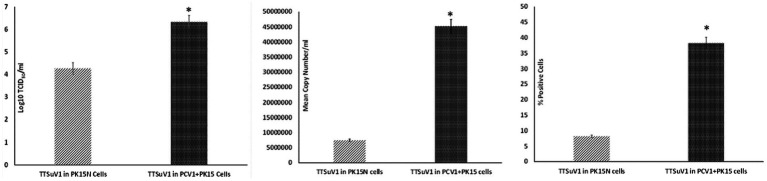
Enhanced replication of TTSuV1 in PCV1 + PK15 cells: PK15N or PCV1 + PK15 cells were transfected with the TTSuV1 dimerized infectious clone. TTSuV1 replication was measured as log_10_TCID_50_ (left), by qPCR (middle) and by flow cytometry (right). Light slanted lines—TTSuV1 dimerized infectious clone transfected in PK15N cells, dark bar—TTSuV1 dimerized infectious clone transfected in PCV1 + PK15N cells. *Significantly different from the PK15N control, *p* ≤ 0.05 (student *t*-test).

To determine if the presence of PCV1 enhances TTSuV1 replication, the dimerized TTSuV1 genome was transfected into PCV1 + PK15 cells as described above. PCV1 free PK15N cells transfected with the dimerized TTSuV1 genome were used as a control. The rescued TTSuV1 was serially passaged in PCV1 + PK15 and PK15N cells six times. The difference in viral replication between treatments was assessed by the TCID_50_ method using the Reed and Muench (1938) formula (Figure 1(left)), by qPCR (Figure 1(middle)) and by flow cytometry (Figure 1(right)).

### 2.5 Replication patterns of TTSuV1 and PCV1 during coinfection *in vitro*

**Figure 2:**
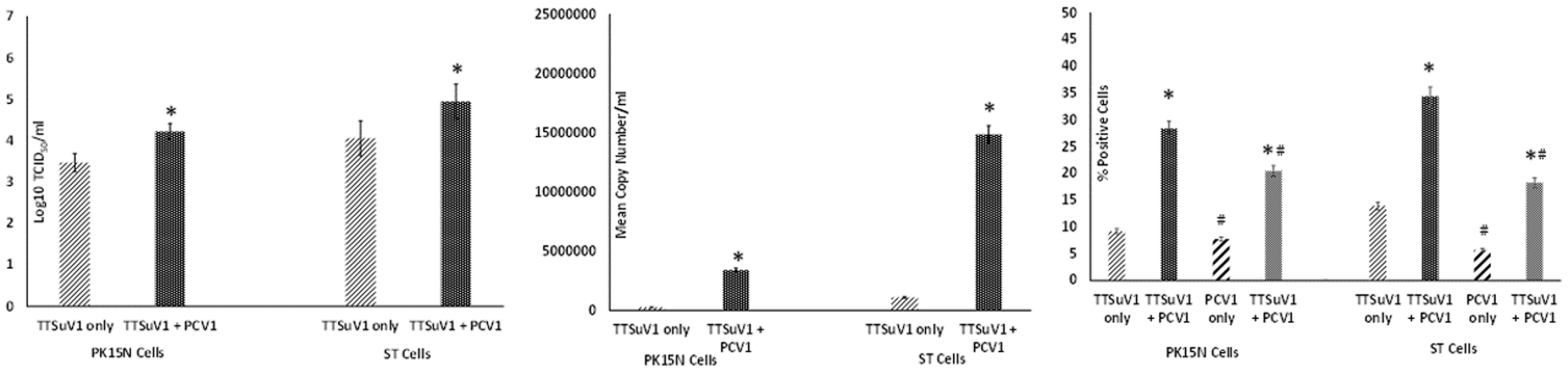
Enhanced replication of TTSuV1 and PCV1 during coinfection *in vitro*: PK15N or ST cells were infected with 1 × 10^3^ TCID_50_ of TTSuV1 only or coinfected with 1 × 10^3^ TCID_50_ TTSuV1 and PCV1 each. TTSuV1 replication was measured as log_10_TCID_50_ (left), by qPCR (middle) and by flow cytometry (right). Light slanted lines—cells infected with TTSuV1 only, dark bar—cells coinfected with TTSuV1 and PCV1, right panel. #Stained with an anti-PCV1 antibody, dark slanted lines—cells infected with PCV1 only, grey dotted bar—cells coinfected with TTSuV1 and PCV1. *Significantly different from the PK15N control, *p* ≤ 0.05 (student *t*-test).

To determine the effects of coinfection with pre-titrated amounts of TTSuV1 and PCV1, PK15N and ST cells were separately cultured in 6-well plates to a confluence of 50%. Cultured cells were infected with 1 mL of PCV1 culture at 1 × 10^3^ TCID_50_/mL. The plates were incubated for 6 h at 37°C in a CO_2_ incubator. The cells were then washed 5 times to remove non-adsorbed PCV1 and then overlaid with 1 mL of TTSuV1 culture which had been resuspended to 1 × 10^3^ TCID_50_/mL. After 24 h, the cells were washed 5× to remove non-adsorbed TTSuV1 and 2 mL of 2% FBS containing DMEM media was added to the cells. The coinfected cells were incubated for 72 h at 37°C in a CO_2_ incubator. Cells infected with TTSuV1 only or PCV1 only and uninfected cells were included as controls. After the 72 h incubation, the plates were frozen, and viral cultures were collected as described above. The difference in viral replication between treatments was assessed by the TCID_50_ method using the Reed and Muench (1938) formula (Figure 2A), by qPCR (Figure 2B) and by flow cytometry (Figure 2C).

### 2.6 Role of PCV1 rep protein in TTSuV1 replication *in vitro*

**Figure 3:**
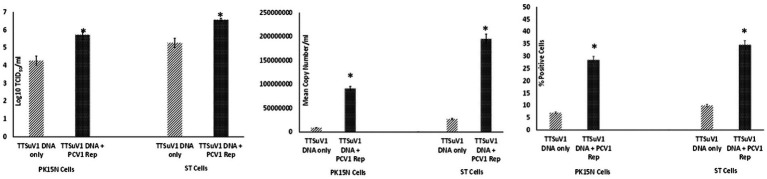
Enhanced replication of TTSuV1 by over-expressed supplementation of PCV1 rep *in vitro*: PK15N or ST cells were transfected with the TTSuV1 infectious clone alone or in combination with a plasmid encoding the PCV1 rep. TTSuV1 replication was measured as log_10_TCID_50_ (left), by qPCR (middle) and by flow cytometry (right). Light slanted lines—cells transfected with the TTSuV1 infectious clone only, dark bar—cells transfected with the TTSuV1 infectious clone and plasmid encoding the PCV1 rep, *Significantly different from the PK15N control, *p* ≤ 0.05 (student *t*-test).

To assess whether the PCV1 rep protein played a role increasing TTSuV1 titers in co-cultures, the procedures described above were repeated with modification. Briefly, PK15N and ST cell-lines were grown to 50% confluence in 25cm^3^ flask. The cultured cells were co-transfected with 2 μg of circularized TTSuV1 genome and 1 μg of plasmid DNA expressing PCV1 replicase, using TransIT-2020 (Mirus Bio, Madison, WI), following the manufacturer’s instructions. Cells transfected with the individual constructs and untransfected cells were used as controls. Effective transfection was assessed by IFA using virus or protein specific antibodies. After a 72 h incubation, the flasks were frozen, and viral cultures were collected as described above. he difference in viral replication between treatments was assessed by the TCID_50_ method using the Reed and Muench (1938) formula (Figure 3A), by qPCR (Figure 3B) and by flow cytometry (Figure 3C).

### 2.7 TTSuV1-specific qPCR

Samples obtained from the experiments described above were tested by a TTSuV1-specific qPCR in duplicate. The qPCR targets a unique region within the ORF1 gene (GenBank accession KT037083, position 1,563–1,565 bps). Briefly, DNA was extracted with the QIAamp DNA Mini Kit (Qiagen, Valencia, CA, United States) following manufacturer’s instructions and eluted into a 50 μL volume. Twenty-five nanogram of template DNA was tested using the QuantiFast Probe PCR Mix (Qiagen United States) with 0.2 μM probe (56 FAM/CACACAACACAGCAGGAA/3IABkFQ) and 0.4 μM primers (5′-TACCCGGCTTTGCTTCGACAGTG-3′ and 5′GCCATAGATTTCTAGCGATCCCAATTGCG-3′). The qPCR program consisted of 95°C for 5 min, followed by 35 cycles of denaturation at 95°C for 15 s, annealing for 57°C for 30 s and extension for 72°C for 30 s, and 5 min holding in a thermocycler (CFX96 Touch, Bio-Rad, Hercules, CA, United States). Each qPCR run included a no template control, plasmid positive control and appropriate standard curve to convert Ct values to copy numbers. The qPCR was optimized to a lowest detection limit of 200 copies/mL (Figures 1B, 2B, 3B).

### 2.8 Flow cytometry

**Figure 4:**
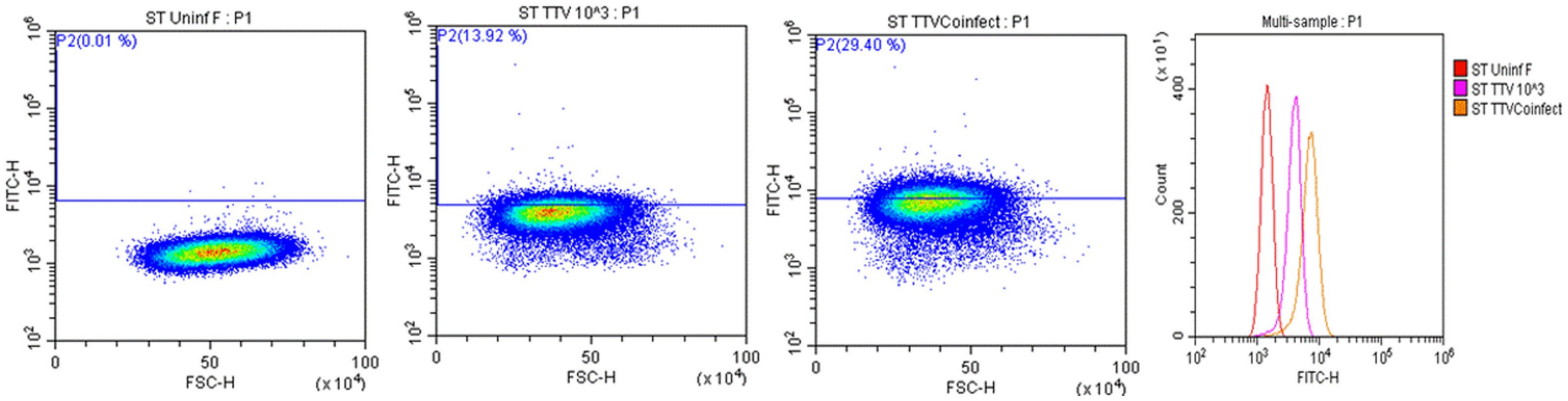
Flow cytometry for the detection of TTSuV1 antigen. Representative images of TTSuV1 infected ST cells stained with TTSuV1-specific anti-peptide rabbit polyclonal antibody. Dot plots—*X* axis—FSC-H, *Y* axis—FITC-H or fluorescent signal due to intracellular TTSuV1 antigen. The horizontal line indicates the gate, numbers in parenthesis indicate the percentage of cells within the gated area. Left dot plot—untreated ST cells, Middle dot plot—ST cells infected with 1 × 10^3^ TCID_50_/mL of TTSuV, right dot plot—ST cells coinfected with 1 × 10^3^ TCID_50_/mL of PCV1 and TTSuV1. On the dot plots, an increase in detection of intracellular antigen is indicated by an upward shift on the *Y* axis. Far right image—overlay histogram depicting the effect of coinfection. *X* axis—FITC H, *Y* axis—counts. Red peak—uninfected ST cells, pink peak—ST cells infected with TTSuV1 only, yellow peak—ST cells coinfected with PCV1 and TTSuV1. On the overlay histogram, an increase in detection of intracellular FITC stained TTSuV1 antigen is indicated by a lateral shift on the *X* axis.

Cells harvested from the above-described experiments were fixed and permeabilized (fixation/permeabilization solution^™^ BD bioscience, Franklin lakes, NJ). Primary antibody working concentrations were optimized by titration. To detect TTSuV1 antigen, intracellular staining was carried out for 45 min at room temperature with rabbit polyclonal TTSuV1 Ab anti-peptide antibody at a 1:200 dilution. Detection was achieved with FITC conjugated anti-rabbit IgG (KPL, Gaithersburg, MD) at a 1:100 dilution in 2% BSA^+^ permeabilization/wash buffer (BD Perm/Wash™, BD bioscience, United States) for 30 min at RT. For the coinfection experiment, cells were also stained with a PCV1-specific polyclonal swine antibody and detected using anti-swine IgG conjugated to FITC (KPL, Gaithersburg, MD) at 1:100 dilution. Stained cells were analyzed in a Cytoflex S flow cytometer (Beckman Coulter, Brea, CA). Single stained and rabbit IgG isotype controls were included in all assays. A high-speed cell sorter was used to count 100,000 events with an appropriate gating strategy. Dead cells, debris and doublets were excluded from analysis. TTSuV1 infected cells were identified by plotting the counts of TTSuV1 antigen positive cells detected by FITC staining against the forward scatter (FSC) height, resulting in an upward shift in fluorescent intensity distribution in dot plots or a lateral shift from the uninfected control cells in a histogram (Figure 4). Data was analyzed using the CytExpert 1.2 software (Beckman Coulter) to determine differences in intracellular fluorescent signal between treatments (Figures 1C, 2C, 3C).

### 2.9 Transmission electron microscopy

**Figure 5:**
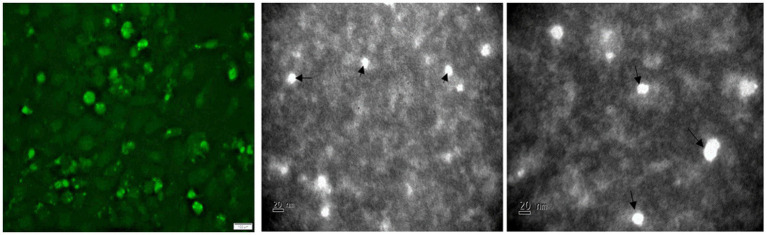
Detection of TTSuV1 virions and PCV1 replicase protein. Left—TTSuV1-specific, intranuclear fluorescence detected by an anti-peptide rabbit polyclonal antibody in an immunofluorescence assay (20× magnification). Middle and right—electron micrographs of TTSuV1 virions after the first passage in ST cells (scale bar = 20 nm).

To confirm the successful assembly and production of recombinant TTSuV1 particles, 50 μL of rescued virus was used to coat a 300-mesh carbon coated palladium grid for 10 min. The coated grid was then negatively stained with 2% phosphotungstic acid (PTA) (Brenner and Horne, 1959) and examined by a JEOL JEM-100CX II transmission electron microscope (Figure 5).

### 2.10 Electrophoretic mobility shift assay

**Figure 6:**
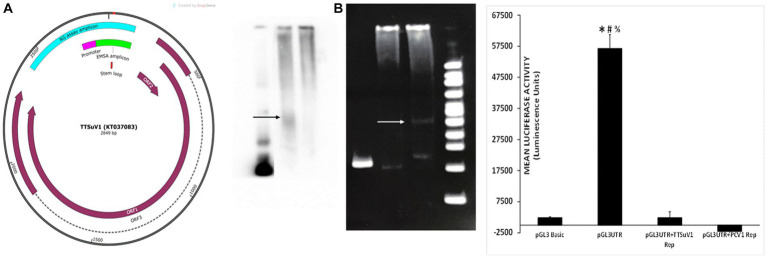
Physical interaction of PCV1 rep with the TTSuV1 untranslated region. Left—genome map of TTSuV1 (Gen Bank accession—KT037083). Brown arrows—identified open reading frames (ORFs), dashed lines—exons, green—amplicon for the EMSA, pink—putative promoter region, teal—amplicon for the reporter gene assay, red—stem loop. Middle panel—electrophoretic mobility shift assay (EMSA). **(A)** Interaction of the biotin labelled TTSuV1 UTR DNA with the PCV1 rep protein detected by chemiluminescence. Arrow shows the upward shift compared to TTSuV1 UTR DNA alone. Lane 1—TTSuV1 UTR DNA only, Lane 2—TTSuV1 UTR DNA hybridized to PCV1 rep, Lane 3—Untransfected negative control cell extract. **(B)** Interaction of the TTSuV1 UTR DNA with the putative replicase protein encoded by TTSuV1 ORF1 as detected by a SYBR green EMSA kit. Arrow shows the upward shift compared to TTSuV1 UTR DNA alone. Lane 4—TTSuV1 UTR DNA only, Lane 5—Untransfected negative control cell extract, Lane 6—TTSuV1 UTR DNA hybridized to TTSuV1 rep. Lane 7—100 bp DNA ladder. Right—reporter gene assay: activity of PCV1 rep and TTSuV1 rep in binding to a promoter located in the TTV UTR. Cells were transfected with either the empty pGL3 basic plasmid, or TTSuV1 UTR region cloned in pGL3 basic (pGL3UTR), or pGL3UTR and TTSuV1 rep expression plasmid or pGL3UTR and PCV1 rep expression plasmid. *X* axis—treatments, *Y* axis—luciferase activity in luminescence units. *Significantly different from pGL3UTR + TTSuV1 rep, *p* ≤ 0.05 (student *t*-test). #Significantly different from pGL3UTR + PCV1 rep, *p* ≤ 0.05 (student *t*-test). #Significantly different from pGL3 basic, *p* ≤ 0.05 (student *t*-test).

The physical interaction between the PCV1 Rep protein and the TTSuV1 intergenic region untranslated region (UTR) (Figure 6 left) was examined by an electrophoretic mobility shift assay (EMSA). Either a biotin-labelling (Pierce 3′ End DNA labelling kit, Thermo Fisher Scientific, Grand Island, NY) or a SYBR based kit (Electrophoretic Mobility-Shift Assay Kit, with SYBR^™^ Green & SYPRO^™^ Ruby EMSA stains, Thermo Fisher Scientific, Grand Island, NY) was used, following manufacturer’s instructions. Briefly, a 210 bp fragment of the TTSuV1 UTR, containing the origin of replication (−76 to +130) and a putative stem-loop signal (Figure 6 left) was amplified by PCR (forward primer: TGATTGGACGGGAGCTCAAGTC and reverse primer: TCCGCTCAGCTGCTCCTGC). The PCV1 and TTSuV1 replicase proteins were expressed by transfection of HEK cells with plasmids encoding the respective proteins. Concentrated nuclear protein was harvested as previously described (Luo et al., 2014). Protein concentration was measured by the BCA method. Extracted protein was immediately aliquoted and stored at −80°C until use.

For hybridization of the DNA to proteins, varying concentrations of DNA were mixed with 5 μL of protein extracts of TTSuV1 or PCV1 replicase protein, in the presence the respective kit hybridization buffers, following manufacturer’s instructions. Poly (d*A*·d*T*) and Poly(d*I*·d*C*) were used as binding competitors in the reaction since the UTR amplicon has high GC content. The reaction was then incubated at room temperature for 20 min. All EMSA reaction products were separated on a 6% Native polyacrylamide gel at 100 V for 1.5 h. TTSuV1 UTR DNA only or untransfected cell extracts incubated with DNA were included as a negative control, while TTSuV1 DNA and TTSuV1 replicase proteins were included as a positive control. The test group included the PCV1 replicase protein and TTSuV1 UTR DNA. The resolved complexes in the gel were immediately transferred onto a nylon membrane, using a semi-dry transfer system with an ice cooling system at 380 mA for 35 min. The membrane was cross-linked before staining. The protein: DNA interaction was detected by either chemiluminescence (Light Shift Chemiluminescence EMSA kit, Thermo Fisher Scientific, Grand Island, NY) or SYBR following the manufacturer’s instructions (Figure 6 middle) and images obtained (FluorChem FC2 Imaging system, Alpha Innotech, San Leandro, CA).

### 2.11 Reporter gene assay

To test the hypothesis that the viral rep proteins can potentially influence viral gene expression by interacting with a TTSuV1 promoter located in the TTSuV1 UTR, a 566 bp segment (Figure 6 right) including the TTSuV1 UTR and the putative promoter which was identified based on published literature (Suzuki et al., 2004; Liu et al., 2016), was cloned into a promoter-less reporter gene system (pGL3 basic, Promega, Madison, WI). The primers used for cloning included CGATgctagcAATCTATGGCCGAGCATGGG and ATGCaagcttTCCGCTCAGCTGCTCCTGC. The cloned region covered −432 to +130 bps adjacent to the TATA box (Figure 6 left). The construct was designated pGL3UTR (pGL3 basic containing TTSuV1 UTR) and validated by sequencing. Vero cells were co-transfected (TransIT-2020, Mirus Bio, Madison, WI), following the manufacturer’s instructions, as described above. The treatments consisted of pGL3UTR + TTSuV1 ORF1, pGL3UTR + PCV1 ORF1. The controls included the pGL3 basic alone (no promoter) as negative control, pGL3UTR as a baseline control, and pGL3UTR^+^ empty V5His plasmid as a background control for the plasmid backbone in which the TTSuV1 and PCV1 rep were expressed. Five replicates of each treatment were tested. After 72 h of incubation at 37°C, luciferase activity was assessed (Bright-Glo kit system, Promega, Madison, WI), following manufacturer’s instructions, and the luminescence readings recorded (Synergy luminometer, Agilent Technologies, Santa Clara, CA). The baseline luciferase activity values for the empty V5HisA backbone of the mammalian expression vector were deducted from the experimental treatments before data analysis (Figure 6 right).

### 2.12 Trans-complementation of PCV2 rep by TTSuV1 rep

**Figure 7:**
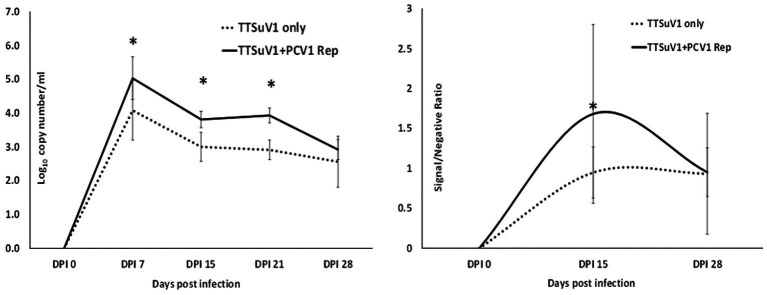
Trans-complementation of the PCV2 replicase protein: immunofluorescence assay (IFA) to detect PCV2 using a PCV2-specific antibody. Effective PCV2 rescue and replication is indicated by apple green, nuclear fluorescence. Blue—DAPI stained nuclei. Top left—uninfected cell control, top middle—cells transfected with the PCV2b ORF1 null mutant, top right—cells transfected with the PCV1 ORF1 (rep) expressing plasmid, bottom left—cells co-transfected with the PCV2b ORF1 null mutant and TTSuV1 dimerized infectious clone, bottom middle—cells co-transfected with the PCV2b ORF1 null mutant and plasmid expressing PCV1 ORF1, bottom right—cells co-transfected with the PCV2b ORF1 null mutant and plasmid expressing TTSuV1 ORF1.

The start codon of the PCV2 replicase protein was deleted by site directed mutagenesis (*Q5 Site*-*Directed Mutagenesis Kit*, Thermo Fisher Scientific, Grand Island, NY), in the backbone of a cloned monomeric genomic copy of PCV2b strain 41,513 (Gen Bank KR816332) (Constans et al., 2015). The deletion was confirmed by sequencing and the functional absence of viral replication was confirmed by IFA by transfecting PK15 cells with the re-circularized mutated PCV2b genomic DNA (Figure 7, top middle). To determine if the mutation could be trans-complemented by the TTSuV1 rep, the mutated PCV2b genomic DNA was co-transfected with either the TTSuV1 ORF1 expression plasmid or the dimerized copy of the TTSuV1 genome, as described above. As PCV1 and PCV2 reps are interchangeable, co-transfection with the PCV1 rep expression plasmid served as a positive control for complementation. Effective rescue and replication of PCV2 due to complementation was assessed by an IFA, using a PCV2 specific antibody (Figure 7). Transfection controls for the TTSuV1 ORF1 and dimerized TTSuV1 genome were checked by IFA using a TTSuV1 specific antibody. Untreated cells were used as a negative control.

### 2.13 Effect of PCV1 replicase on TTSuV1 replication in mice

**Figure 8:**
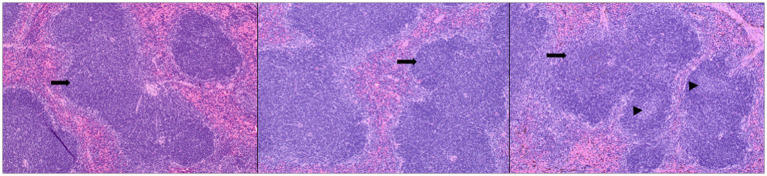
Replication of TTSuV1 in mice. Left—copy numbers of TTSuV1 DNA as detected in whole blood by a TTSuV1-specific qPCR assay. *X* axis—days post infection, *Y* axis—log_10_ copy number/mL. Solid line—mean values for mice administered TTSuV1 infectious clone and plasmid encoding the PCV1 replicase. Dotted line—mean values for mice administered TTSuV1 infectious clone alone. Right—antibody responses in mice. TTSuV1-specific IgG responses measured by an ELISA. Solid line—mean values for mice administered TTSuV1 infectious clone and plasmid encoding the PCV1 replicase. Dotted line—mean values for mice administered the TTSuV1 infectious clone alone. *X* axis—days post infection, *Y* axis—signal/negative ratio. *Significantly different from the single infection group, *p* ≤ 0.05 (student *t*-test).

All animal experimentation was carried out in compliance with the N. Dakota State University IACUC regulations. To determine if the presence of PCV1 rep will enhance TTSuV1 replication *in vivo*, 24 2 weeks-old, male and female C57/BL6 mice were administered treatments as follows: Group I—300 μL PBS, 50 μL i/n and 250 μL i/m, *N* = 4, Group II—50 μg of plasmid DNA encoding the PCV1 rep in a 300 μL volume, 50 μL i/n and 250 μL i/m, *N* = 4, Group III—50 μg of plasmid DNA encoding the TTSuV1 dimerized genome in a 300 μL volume, 50 μL i/n and 250 μL i/m, *N* = 8, Group IV—50 μg of plasmid DNA encoding the PCV1 rep and TTSuV1 dimerized genome each in a total of 300 μL volume at 50 μL i/n and 250 μL i/m, *N* = 8. Group III and Group IV mice were administered the TTSuV1 dimerized plasmid 24 h after administration of the PCV1 rep plasmid. The mice were observed daily for any signs of physical abnormalities. Whole blood was collected on day 0, 7, 15, 21 and 28 post inoculation to assess TTSuV1 genome copy numbers by qPCR. Serum was collected on day 0, 15, 28 post infection to assess antibody responses to TTSuV1 by ELISA. Samples were assessed in duplicate by qPCR using DNA extracted with the QIAamp DNA blood mini-Kit (Qiagen, Valencia, CA, United States), essentially as described above (Figure 8—left). Half the number of mice in each group were euthanized on day 15 and the remaining mice on day 30 post exposure. Liver, kidney, spleen, heart, lungs, large intestine, and ileum were fixed in 10% formaldehyde for histopathological analysis.

### 2.14 TTSuV1 ELISA

The measurement of antibody responses to TTSuV1 was carried out essentially as described previously (Ssemadaali et al., 2016). Briefly, 96 well ELISA plates (High bind microplates, Corning^®^, NY) were coated with 50 μL of purified recombinant TTSuV1 ORF2 antigen (1:100,000 dilution) in carbonate coating buffer (pH 9.6), overnight at room temperature. The coated plates were washed with PBST and blocked with a commercial block (General block, ImmunoChemistry Technologies, Bloomington, MN) containing 2% BSA and 2% normal goat serum for 2 h at 37°C. The experimental mouse sera were diluted to 1:10 in PBST containing 2% BSA dilution and 50 μL volumes were added to the plate. The plates were incubated for 1 h at 37°C. Detection was achieved using a goat anti-mouse HRPO-conjugate (KPL, Gaithersburg, MD) at a 1:2500 dilution for 45 min at 37°C following incubation with the TMB substrate (KPL, Gaithersburg, MD). The reaction was stopped after 5 min by adding 1 M HCl solution. Plates were read at 450 nm using ELISA plate reader (Elx800 reader, BioTek Instruments, Inc., Winooski, VT). Samples were assessed in three independent assays in duplicate (minimum 4 values each as some samples were exhausted). Day 0 samples were used to obtain the mean baseline for negative samples. Data across each independent assay was normalized using the negative control. The optical density values were expressed as a signal to negative ratio after reduction of the background (Figure 8—right).

### 2.15 Assessment of histopathological lesions

**Figure 9:**
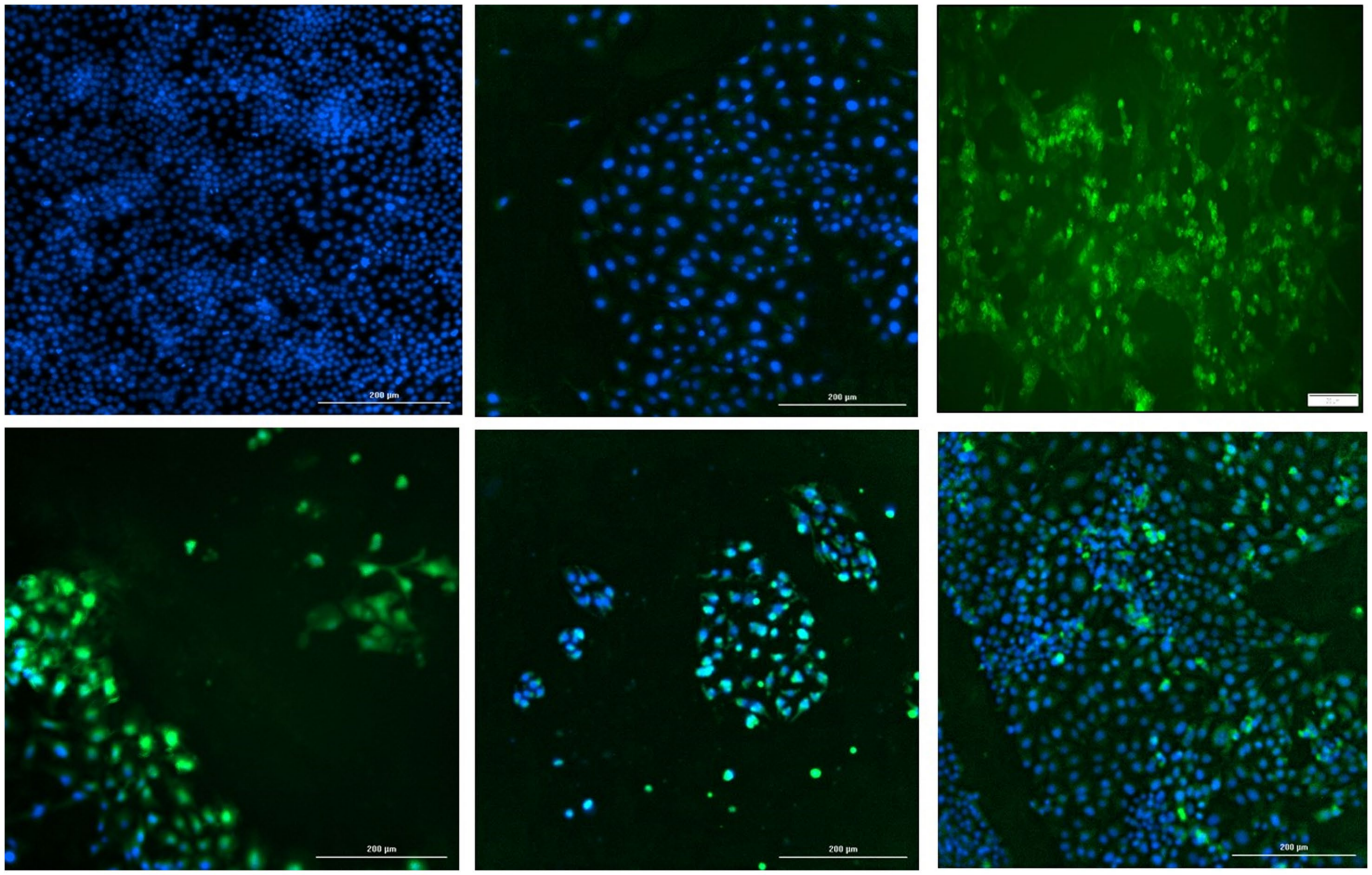
Splenic lesions in infected mice: representative images of H&E-stained sections of mouse spleen. Left—untreated mice, middle—mice administered the TTSuV1 infectious clone alone demonstrating mild lymphoid hyperplasia, right—mice administered both the TTSuV1 infectious clone and PCV1 rep encoding plasmid DNA demonstrating moderate lymphoid hyperplasia. No differences in extramedullary hematopoiesis were detected. Arrows—white pulp, arrowheads—germinal center development. magnification-100×.

The mice were examined for any gross changes to organs during necropsy. Sections of lung, liver, spleen, pancreas, kidney, and intestines were stained by hematoxylin and eosin following the standard operating procedures of the *N*. Dakota State University Veterinary Diagnostic Laboratory. The sections were scored for microscopic lesions in a blinded fashion by a board-certified pathologist. Lymphoid hyperplasia and extramedullary hematopoiesis in the spleen were assessed. The scoring system used to grade lymphoid hyperplasia was as follows: Marked decrease =2, moderate decrease =2, mild decrease =3, normal =4, mild increase =5, moderate increase =6, marked increase =7. Extramedullary hematopoiesis was scored as follows: normal =1, mild =2, moderate =3, marked =4 (Figure 9).

### 2.16 Statistical analysis

Data analysis was carried out using Microsoft excel or Minitab 19 (Minitab, State College, PA). Following assessment for normal distribution, significance levels were set at *p* < 0.05 for all tests. The ELISA, qPCR, luminescence and histology data were analyzed by the student’s *t*-test. The figures and graphs included depict average or consolidated values with standard deviations and statistical significance for differences between test groups.

## 3 Results

### 3.1 The presence of PCV1 or PCV1 rep protein enhances TTSuV1 replication

To determine whether PCV1, or specifically the PCV1 rep protein, can increase TTSuV1 titers in cell culture, PK15N or ST cells were either co-infected with PCV1 and TTSuV1 or co-transfected with a recombinant TTSuV1 infectious clone and plasmid expressing the PCV1 rep. Three different quantification techniques were used to quantify virus; log_10_TCID_50_ to measure replicative TTSuV1 (Figure 1 left, Figure 2 left, and Figure 3 left), a TTSuV1-specific qPCR to measure viral genome copy numbers (Figure 1 middle, Figure 2 middle, and Figure 3 middle) and flow cytometry to measure intracellular TTSuV1 antigen (Figure 1 right, Figure 2 right, and Figure 3 right).

Transfection of the dimerized TTSuV1 genome in PCV1 + PK15 cells resulted in a significantly higher replication of TTSuV1 compared to transfection in PK15N cells, indicating that the presence of PCV1 supported the replication of TTSuV1 (Figure 1). Further, the viability of the rescued TTSuV1 was sustained without a loss of titer for 10 serial passages in PCV1 + PK-15 cells but not in the PCV1 negative PK15 N cells (data not shown), as previously observed by others (Huang et al., 2012). To further confirm the results, PK15N and swine testicular (ST) cells were coinfected with pre-titrated and equal amounts of PCV1 and TTSuV1. The coinfected cells had significantly higher TTSuV1 titers compared to singly infected cells (Figure 2). To specifically determine if the PCV1 rep played a role in enhanced TTSuV1 replication in coinfections, possibly via trans-replicase activity, the PCV1 replicase protein was transiently over-expressed in PK15N and ST cells, in combination with the dimerized TTSuV1 genome. Consistent with previous observations, higher levels of TTSuV1 replication were detected in cells co-transfected with the PCV1 rep and TTSuV1 infectious clone compared to cells singly transfected with the TTSuV1 infectious clone alone (Figure 3). The observed results were consistent across the three different quantification techniques, i.e., log_10_TCID_50_, qPCR and flow cytometry. Overall, ST cells supported higher levels of TTSuV1 replication than PK-15 cells across the treatment conditions. Reciprocally, measurement of PCV1 intracellular antigen by flow cytometry showed that PCV1 antigen detection was also significantly higher in the presence of TTSuV1 (Figure 2 right, treatments indicated by a #). Untransfected or uninfected cells remained clear of specific signals for all experiments. Representative images for data acquisition by flow cytometry are provided in Figure 4. The specificity of the TTSuV1 anti-peptide rabbit antibodies used was independently verified with previously validated antibodies (Huang et al., 2011) (Figure 5 left). The rescue of structurally intact TTSuV1 was further confirmed by the presence of approximately 20-25 nm sized icosahedral particles by electron microscopy (Figure 5 middle and right).

### 3.2 The PCV1 replicase protein interacts with the TTSuV1 UTR region

Exploration of the physical interaction of the PCV1 replicase protein with the intergenic TTSuV1 UTR region (Figure 6 left) by an electrophoretic mobility shift assay (EMSA), showed that the PCV1 rep colocalized with the TTSuV1 UTR, as indicated by an upward shift in the sample containing both the TTSuV1 UTR DNA and PCV1 rep protein when compared to the sample with the TTSuV1 UTR DNA alone (Figure 6A middle). As expected, the TTSuV1 rep positive control and was found to bind to the TTSuV1 UTR DNA, as indicated by an upward shift (Figure 6B middle) compared to the TTSuV1 DNA only control.

As the TTSuV1 UTR is believed to contain a putative promoter (Figure 6 left), the possible interaction of the PCV1 and TTSuV1 rep proteins with the promoter was explored using a luciferase-based reporter gene assay. When the cloned reporter gene constructed containing the UTR fragment (pGL3UTR) was transfected into Vero cells, strong promoter activity was detected luciferase activity (Figure 6 right), while transfection of the empty control pGL3 basic plasmid did not. However, when a mammalian protein expression plasmid, encoding either the PCV1 rep or TTSuV1 ORF1 proteins was co-transfected along with the pGL3UTR plasmid, the promoter activity was completely abrogated, indicating that both TTSuV1 and PCV1 rep proteins bound with very high affinity to the UTR DNA, out competing the binding of the cellular transcription factors that stimulated the reporter gene expression (Figure 6 right).

### 3.3 The TTSuV1 ORF1 product trans-complements a PCV2 replicase null mutant

To determine if the TTSuV1 and PCV reps can function interchangeably, the start codon of the rep gene was deleted in a PCV2b infectious clone by site directed mutagenesis. As expected, deletion of the ATG of the rep gene resulted in abrogation of viral replication in cells transfected with the mutated PCV2b infectious clone, as assessed absence of green, fluorescent signals by an IFA (Figure 7 top middle). However, co-transfection of the mutated PCV2b infectious clone with either the dimerized TTSuV1 infectious clone (Figure 7 bottom left), or a plasmid encoding the PCV2 ORF1 (Figure 7 bottom middle) or a plasmid encoding the TTSuV1 ORF1 (Figure 7 bottom right) trans-complemented the silenced PCV2b rep and resulted in successful rescue of PCV2b as evidenced by apple green nuclear fluorescence in IFAs.

### 3.4 PCV1 replicase increases TTSuV1 genome copy numbers in mice

To determine if the presence of PCV1 rep will enhance TTV replication *in vivo*, mice were administered the TTSuV1 infectious clone alone or in combination with the PCV rep encoding plasmid. A comparison of the TTSuV1 genome copy numbers by qPCR showed that mice in the dually exposed group had higher copy numbers of TTSuV1 DNA in all the time points tested. The difference was statistically significant at days post exposure 7, 15 and 21 but not at day 28. TTSuV1 DNA was not detected in the control mice administered PBS or DNA encoding the PCV1 replicase alone (Figure 8 left). When antibody responses against TTSuV1 were measured by ELISA the rate and magnitude of seroconversion was greater at 15 days post-exposure in the dually exposed mice when compared to the mice inoculated with TTSuV1 DNA alone (Figure 8 right). Antibody levels in both experimental groups were similar by 28 days, correlating with the kinetics of viral replication observed by qPCR (Figure 8). The expression of the PCV1 rep protein in mice was verified by the detection of antibody responses against the PCV1 rep using an immunofluorescence assay (data not shown).

The primary observable microscopic lesions in the experimental mice consisted of splenic lymphoid hyperplasia (Figure 9). The mean scores were slightly higher in the co-inoculated group (DPI 15–5.5 ± 0.58, DPI 30–5.67 ± 0.58) compared to the singly inoculated group (DPI 15–5.25 ± 0.50, DPI 30–5.25 ± 0.50). However, the differences were not statistically significant. Consistent microscopic lesions or significant gross changes were not observed in other major organs.

## 4 Discussion

While the extensive epidemiological association of TTVs with a variety of infectious and non-infectious disease conditions is well documented in published literature, current understanding of the mechanisms involved is limited, likely due to the difficulty in culturing TTVs in the laboratory and a lack of standardized animal models (Kekarainen and Segales, 2012, Spandole et al., 2015, Webb et al., 2020). We and others have observed that transfection of TTSuV genomic DNA in cells results in viral protein production and infection, but there is progressive loss of viability of the rescued virus (Huang et al., 2012; Singh and Ramamoorthy, 2016a,b). The serendipitous observation that high TTSuV1 titers were produced when a dimerized TTSuV1 infectious clone was accidentally transfected into PCV1 + PK15 cells instead of the PCV1 negative PK15N cells, prompted the exploration of synergies in the mechanisms of replication of PCVs and TTSuV1 in this study.

Coinfection of adeno associated virus (AAV), a dependovirus, with its helper viruses is reported to reduce the helper virus’s replication (Meyers et al., 2001). However, in this study, the positive effect on replication appeared to be reciprocal for PCVs and TTSuV1 as increased PCV1 signals were observed in coinfected cells by flow cytometry (Figure 2C). The intracellular TTSuV1 or PCV1 antigen measured by flow cytometry very likely represented viable virus and similar flow cytometry-based strategies have been used to measure viral replication for other viruses (Shen et al., 2002). The trend of observations being consistent between the 3 different quantification methods used (Figures 1–3) provides robust validation for the conclusion that PCV1, and specifically PCV1 rep, positively influences TTSuV1 replication.

Similar to our findings that the PCV1 replicase protein interacts physically with the TTSuV1 UTR (Figure 6), the helicase primase complex and ssDNA binding proteins of helper herpes simplex viruses bind to the AAV inverted terminal repeats (ITRs) to promote AAV DNA replication (Weindler and Heilbronn, 1991). Further, a human herpes virus 6 (HHV6) protein is reported to act as a homolog to the AAV rep protein (Thomson et al., 1994). Rolling circle amplification (RCR) of circular DNA genomes is achieved via the nicking, helicase, ATPase and endonuclease activity of replicase proteins (Wawrzyniak et al., 2017). While we demonstrate that the PCV1 rep physically interacts with to the TTSuV1 UTR DNA (Figure 6), performing mutation and complementation analysis to determine which of these functions is executed or facilitated by the non-cognate rep protein or the understand the exact mechanistic nature of the interaction is not within the scope of this study.

Transcriptional regulation is another recognized mechanism by which coinfecting viruses can influence each other’s replication and pathogenesis. For example, adenoviral proteins are known to bind to an AAV promoter upregulating the transcription of the AAV rep proteins (Chang et al., 1989). The full-length PCV1 rep represses its own transcription initiation, whereas the spliced *Rep*′ protein does not (Mankertz et al., 2003). While the transcriptome of TTVs is yet to be fully understood, a TTV minimal promoter has been previously mapped to the intergenic UTR region in human TTVs (Figure 6 left) (Suzuki et al., 2004; Liu et al., 2016). While described data confirms the presence of the promoter in TTSuV1 (Figure 6 right), the exact TTSuV1 UTR residues involved in binding to the rep proteins are not known and were not identified in this study. As the putative promoter region was present in both the EMSA and reporter gene amplicons (Figure 6 middle and right), it is reasonable to infer that PCV or TTSuV1 rep proteins may not have transcription factor like functions but rather bind to the UTR region with very high affinity to promote viral DNA replication. Based on other studies showing that the TTSuV1 UTR region also contains regulatory and binding sequences for NF-kB, SP-1, AP-2, ATF/CREB and that the TTV ORF2 product downregulates the NF-kB (Zheng et al., 2007), IFN-β and IL-13 (Singh and Ramamoorthy, 2016a,b). It is possible that other indirect mechanisms may also be involved in TTVs and PCVs promoting each other’s replication.

As the TTSuV1 ORF1 is believed to encode a bifunctional protein that serves both as the capsid and has replicase activity, deletion of the start codon of the TTSuV1 ORF1 would result loss of both capsid and rep functions. Hence, this approach could not be used to assess trans-complementation functions. Hence, a rep deficient PCV2b mutant which could potentially be trans-complemented by the TTSuV1 ORF1 product was developed (Figure 7). Besides sharing conserved rep proteins, circoviruses share a conserved nonanucleotide sequence required for RCR with other CRESS viruses and even related plant viruses from the *Geminiviridae* and *Nanoviridae* families. Some monopartite begomoviruses which belong to *Geminivirdae* require beta satellite nanoviruses to induce overt disease. The beta satellite viruses are promiscuous in rep binding or ATPase interactions as they associate with several cognate and non-cognate helper *geminiviruses* via their trans-acting rep binding sites (Zhang et al., 2016). Given the genetic relatedness of ssDNA viruses, it is highly likely that relaxed specificity of the rep binding motifs for TTVs and PCVs can allow for similar trans-acting binding with structurally related replicases from other viruses.

Rodent TTVs have been previously described in rats. Administration of TTV positive human blood resulted in the detection of TTV DNA in the blood and tissues of the exposed mice for several days (Isaeva and Viazov, 2002; Xiong et al., 2018). As TTVs are not usually pathogenic, overt clinical signs or severe lesions were not expected or observed in the experimental mice in this study (Figure 9). As active viral DNA replication occurs early in infection, it is likely that the maximum load of TTSuV1 DNA was observed at day 7 post-exposure (Figure 8 left). Although the detection of TTSuV1 DNA by itself may not mean that productive viral infection occurred, the observation that seroconversion occurred between days 0–15 post-exposure (Figure 8 right) in a pattern consistent with the qPCR data and moderate splenic lesions were observed in dually exposed mice is suggestive of productive TTSuV1 infection. A more detailed characterization of TTV infection in mice was not undertaken and is the focus of future studies. While published epidemiological and experimental studies show that TTVs exacerbate PCV2 clinical signs, the primary hypothesis tested in this study is that the replicase protein of PCVs has trans-replicase activity for TTVSuV1. The replicase proteins of PCV1 and PCV2 are highly conserved and exchangeable. Using the PCV1 system enabled us to study the effects of the rep on TTSuV1 without any possible confounding pathogenic effects due to PCV2 in the mouse model.

In conclusion, data from this study suggests that an overlap in viral DNA replication pathways can lead to increased viral replication and possibly disease manifestations, thus providing novel insight into the complex molecular interactions between TTVs and PCVs in coinfections. The current study also has implications for developing better laboratory culture methods and *in vivo* models for TTV’s. With the current explosion in the discovery of small DNA viruses due to metagenomic sequencing and emphasis on understanding how the microbiome and virome contribute to health and disease, improved knowledge regarding how viruses interact in coinfections can be critical to developing better tools for prevention.

## Data availability statement

The datasets presented in this study can be found in online repositories. The names of the repository/repositories and accession number(s) can be found in the article/supplementary material.

## Ethics statement

The animal study was approved by North Dakota State University Institutional Animal Care and Ethics Committee. The study was conducted in accordance with the local legislation and institutional requirements.

## Author contributions

MS: Data curation, Investigation, Methodology, Writing – review & editing. M-TI: Data curation, Investigation, Methodology, Writing – original draft, Writing – review & editing. WF: Investigation, Methodology, Writing – review & editing. ZA: Data curation, Formal analysis, Investigation, Methodology, Writing – review & editing. BW: Data curation, Formal analysis, Investigation, Methodology, Writing – review & editing. SR: Conceptualization, Funding acquisition, Investigation, Methodology, Resources, Supervision, Writing – original draft, Writing – review & editing.
